# Activation of Hepatic Lipase Expression by Oleic Acid: Possible Involvement of USF1

**DOI:** 10.3390/nu1020133

**Published:** 2009-10-29

**Authors:** Diederik van Deursen, Marije van Leeuwen, Deniz Akdogan, Hadie Adams, Hans Jansen, Adrie J.M. Verhoeven

**Affiliations:** 1 Dept. Biochemistry, Cardiovascular Research School (COEUR), Erasmus MC, PO Box 2040, 3000 CA Rotterdam, The Netherlands; Email: d.vandeursen@erasmusmc.nl (D.v.D); m.vanleeuwen@viroscope.com (M.v.L.); 2 Dept. Clinical Chemistry, Cardiovascular Research School (COEUR), Erasmus MC, PO Box 2040, 3000 CA Rotterdam, The Netherlands; Email: jan82106@planet.nl (H.J.)

**Keywords:** monounsaturated fatty acids, sterol-responsive element binding protein SREBP2, upstream stimulatory factor USF1, *LIPC*, transcriptional regulation

## Abstract

Polyunsaturated fatty acids affect gene expression mainly through peroxisome proliferator-activated receptors (PPARs) and sterol regulatory element binding proteins (SREBPs), but how monounsaturated fatty acids affect gene expression is poorly understood. In HepG2 cells, oleate supplementation has been shown to increase secretion of hepatic lipase (HL). We hypothesized that oleate affects HL gene expression at the transcriptional level. To test this, we studied the effect of oleate on HL promoter activity using HepG2 cells and the proximal HL promoter region (700 bp). Oleate increased HL expression and promoter activity 1.3–2.1 fold and reduced SREBP activity by 50%. Downregulation of SREBP activity by incubation with cholesterol+25-hydroxycholesterol had no effect on HL promoter activity. Overexpression of SREBP2, but not SREBP1, reduced HL promoter activity, which was effected mainly through the USF1 binding site at -307/-312. Oleate increased the nuclear abundance of USF1 protein 2.7 ± 0.6 fold, while USF1 levels were reduced by SREBP2 overexpression. We conclude that oleate increases HL gene expression via USF1. USF1 may be an additional fatty acid sensor in liver cells.

## 1. Introduction

Polyunsaturated fatty acids (PUFAs) affect gene expression through interaction with specific transcription factors, notably peroxisome proliferator-activated receptors (PPARs) and sterol regulatory element binding proteins (SREBPs) [1,2]. PUFAs are ligands for PPARs, and binding results in the formation of an active transcription factor [3]. SREBP1 and −2 are cholesterol-sensitive transcription factors that are involved predominantly in regulation of fatty acid synthesis and cholesterol homeostasis, respectively [4]. Immature SREBP proteins are present in the endoplasmic reticulum membrane; upon transport to the Golgi, the transcriptionally active *N*-terminal part of the protein (nSREBP) is released by proteolytic cleavage [5]. PUFAs are shown to modulate both the synthesis and maturation of the SREBPs [1,4,6]. In short-term feeding experiments, the mRNA profile of mouse liver almost completely overlapped between PUFAs and specific PPARα agonists, suggesting that PUFAs mainly act through PPARα [7]. PUFAs also reduce SREBP activity or nSREBP1 protein in rat and mouse liver *in vivo* [8,9,10,11], rat hepatocytes [10,11] and human HepG2 hepatoma cell lines [6,12]. 

Monounsaturated fatty acids (MUFAs), notably oleate, are the most abundant fatty acids in human plasma. Compared to PUFAs, the effect of MUFAs on liver gene expression is relatively small [7]. How gene expression is affected by MUFAs is poorly understood. It is assumed that like PUFAs, MUFAs signal through PPARs and SREBPs, but short-term feeding of mice with triolein showed relatively limited overlap in mRNA profile with PUFAs or with PPARα agonists; of the 114 genes affected by triolein feeding, 65 (57%) were unique to triolein, whereas of the 519 genes affected by PUFAs, only 89 (17%) were unique for PUFAs [7]. In addition, oleate is much less effective than PUFAs in suppressing SREBP activity and nSREBP1 protein levels [6,12]. This suggests that MUFAs may affect gene expression through mechanisms other than PPARs and SREBPs. 

We are interested in how the human hepatic lipase (HL) gene is upregulated by oleic acid. Hepatic lipase (EC 3.1.1.3) is an extracellular enzyme present on cell membranes in liver sinusoids, where it has an important role in plasma lipid and lipoprotein metabolism [13,14,15]. Post-heparin plasma HL activity is elevated in type 2 diabetes [16], increases with the HOMA-index, a measure of insulin resistance, in non-diabetic males [17], and increases with visceral fat mass [18,19]. Hence, HL activity appears to be high under conditions with increased supply of fatty acids to the liver. In rats, diets rich in either saturated fats [20] or fish oil [21] reduced post-heparin plasma HL activity, but the effect of selective MUFA enrichment has not been reported. HepG2 cells supplemented with oleate showed increased HL expression [22,23], which is due at least in part to increased transcription of the HL gene [23]. In human studies, treatment with PPARα agonists minimally elevated HL activity [24,25], whereas in rats fenofibrate strongly suppressed HL expression [26]. It seems unlikely therefore that the effect of oleate on HL expression is explained by activation of PPARα. Treatment with statins, which act predominantly through elevation of SREBP activity, consistently results in reduction of HL activity [16,27]. In HepG2 cells, atorvastatin as well as forced expression of nSREBP2 reduced HL secretion and HL gene transcription [23]. However, feeding rats a cholesterol-enriched diet, which suppresses SREBP activity, was also reported to reduce HL expression [28]. Our previous studies using HepG2 cells suggested that SREBP2 interferes with the sensitivity of the HL gene to upregulation by Upstream Stimulatory Factors (USFs) [23]. USF1 and 2 are ubiquitous transcription factors involved in the regulation of many genes including the insulin-responsive and lipogenic enzymes expressed in liver [29,30]. Binding of USFs to their cognate site in the HL promoter region strongly increased its transcription [31,32]. Overexpression of nSREBP2 in HepG2 cells appeared to abolish this responsiveness to USFs [23]. Hence, the HL gene may be an indirect target of the SREBPs.

In the present paper, we tested the hypothesis that HL gene expression is affected by oleate at the level of transcription. As a model, we used the proximal promoter region of the HL gene upon transient transfection of HepG2 cells. Our results show that supplementation of HepG2 cells with oleate increases the nuclear abundance of USF1, which may at least in part explain the stimulatory effect of oleate on HL promoter activity [33]. 

## 2. Results and Discussion

### 2.1. Oleate Increases HL Expression and Down-Regulates SREBP Activity

**Figure 1:**
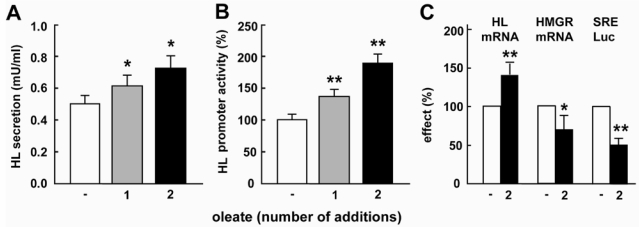
Oleate increases HL expression and down-regulates SREBP activity. HepG2 cells were incubated for 48h without further additions (-), with BSA-bound oleate added once at the start of the incubation (1) or with oleate added both at the start and again after 24h (2). At the end of the incubation, secretion of HL activity (A), HL-685 luciferase activity (B) and HL mRNA (C) was determined. In parallel, HMG-CoA reductase (HMGR) mRNA and SRE-luciferase (SRE-Luc) was measured (C). (n = 3-5; *: *P* < 0.05 and **: *P* < 0.01 vs. control).

When HepG2 cells were supplemented with oleate (1 mM BSA-bound), and then incubated for 48 h, secretion of HL activity (Figure 1A) and luciferase activity of the HL-685 promoter construct (Figure 1B) were significantly increased. 

By this time however, oleate was no longer detectable in the extracellular medium [23]. When an extra addition of oleate was given after 24 h, secretion of HL activity and HL-685 luciferase activity further increased (Figures 1A and B), suggesting a dose-response relationship. HL mRNA increased in parallel to HL secretion and HL-685 luciferase activity (Figure 1C). Simultaneously, SRE-luc activity and HMG-CoA reductase (HMGR) mRNA, an SREBP2 target gene, were significantly suppressed by oleate supplementation (Figure 1C). 

### 2.2. HL Promoter Activity Is Down-Regulated by SREBP-2 but not by SREBP-1

**Figure 2:**
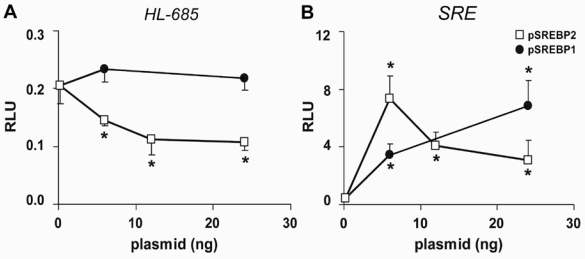
Effect of nSREBP2 and nSREBP1 on HL-685 promoter activity. HepG2 cells were transfected with the plasmidsHL−685 (A) or SRE-luc (B), and the indicated amounts of pSREBP1 or pSREBP2 (open and closed symbols, respectively). Relative luciferase activity (RLU) was determined after 48h (n = 3; *: *P* < 0.05 compared to control).

To test whether the HL gene is a target of SREBP1 or SREBP2, we transfected HepG2 cells with pSREBP1 and pSREBP2, which encode the nuclear form of SREBP1 and SREBP2. The activity of HL-685 was dose-dependently down-regulated by pSREBP2 up till 50% (Figure 2A). HL promoter activity was not significantly affected by pSREBP1. Qualitatively similar results were obtained with the HL-325 construct (not shown). In parallel, both pSREBP2 and pSREBP1 increased SRE-luc activity 8-14 fold (Figure 2B). Hence, HL promoter activity is down-regulated by SREBP2, but not by SREBP1.

**Figure 3:**
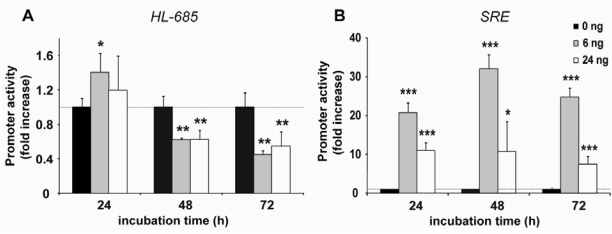
nSREBP2 overexpression suppresses HL-promoter activity time-dependently. HepG2 cells were transfected with HL−685-luc (A) or SRE-luc (B), either without or with6 or 24 ng pSREBP2 (dark, grey and white bars, respectively). Luciferase activities were determined at the incubation times indicated, and expressed as fold increase relative to the no-pSREBP2 control; (n = 4; *: *P* < 0.05, **: *P* < 0.01, and ***: *P* < 0.001 vs. control).

To determine the correlation between SREBP activity and down-regulation of the HL promoter, we determined luciferase activity at different time points after co-transfection. Suppression of HL–685 activity was only apparent at 48 and 72 h with both 6 and 24 ng of pSREBP2 (Figure 3A). In fact, after 24h, HL promoter activity was slightly increased. In contrast, SRE luc activity was already strongly and maximally increased after 24h, and remained high thereafter (Figure 3B). 

When HepG2 cells were incubated with cholesterol+25-hydroxycholesterol, SREBP activity was strongly suppressed to 8 ± 3% of control (n = 6, *P* < 0.001). The activity of the HL–685 construct was not significantly affected (109 ± 40%, n = 7). Apparently, lowering of SREBP activity *per se* is not sufficient to increase HL promoter activity. Combined with the delayed response of HL promoter activity to overexpression of nSREBP2, this suggests that the HL gene is not a direct target of SREBP2. This can also be deduced from the oligonucleotide microarray data from Horton *et al.* [34], who showed that HL mRNA was reduced in SREBP1 and SREBP2 transgenic mice but not increased in SREBP-cleavage activating protein (SCAP) knockout mice. 

**Figure 4:**
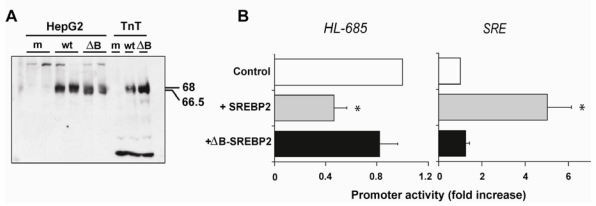
The DNA binding domain of nSREBP2 is required for suppression of HL-685. (A) HepG2 cells were transfected in a 6-wells plate with 1 µg of empty pcDNA3 vector (m = mock), 1 µg wildtype (wt) or 1 µg ΔB-SREBP2. SREBP2 protein was detected in nuclear extracts by immunoblotting with RS004 anti-SREBP2. TnT extracts with *in vitro* made SREBP2 or ΔB-SREBP2 protein (all 0.5 μl) were run in parallel (TnT). Molecular sizes are indicated in kDa. (B) HepG2 cells were co-transfected in a 24-wells plate with 24 ng pSREBP2 or ΔB-SREBP2, and either HL-685-(left panel) or SRE-luc (right panel). Data are expressed as fold increase with respect to mock transfection (control); (n = 4; *: *P* < 0.05 vs. control).

### 2.3. Down-Regulation of the HL Promoter by SREBP2 Requires an Intact DNA Binding Domain

We then constructed the ΔB-SREBP2 expression plasmid, which encodes a mutant form of nSREBP2 lacking the basic, DNA binding domain [35,36]. Wildtype and ΔB-SREBP2 plasmids induced similar amounts of the protein product when expressed *in vitro* and upon transfection into HepG2 cells (Figure 4A). In contrast to the wildtype protein, the mutant protein was not detectable on immunoblots using the monoclonal anti-SREBP2 from IgG-1D2 hybridoma raised against aa 48–403 of human SREBP2, but both proteins were detectable by polyclonal RS004 anti-SREBP2. Co-transfection of HepG2 cells with up till 100 ng of the ΔB-SREBP2 plasmid did not significantly affect the activity of HL-685 and SRE-luc (Figure 4B). Hence, the DNA binding domain of SREBP2 is required for the down-regulation of HL promoter activity.

### 2.4. nSREBP2 Exerts Its Effect Predominantly via the -307/-312 Region of the HL Promoter

**Figure 5:**
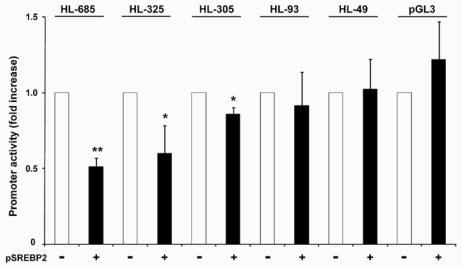
Effect of 5′-deletions on the down-regulation of HL-685 activity by nSREBP2. HepG2 cells were co-transfected with the indicated HL constructs (or empty pGL3-basic) without (-) or with (+) 6 ng pSREBP2. Luciferase activity was determined after 72h. The activity without pSREBP2 was taken as 1.0 (n = 3–4; *: *P* < 0.05, ** *P* < 0.01 vs. control).

To identify which part of the HL promoter is responsible for the down-regulation by SREBP2, a series of 5’-deletions was generated from the HL-685 promoter fragment. As shown in Figure 5, overexpression of SREBP2 caused a 40% reduction in activity of HL-325, similar to the parent construct. The HL-305 construct was only slightly, but significantly, down-regulated by SREBP2, whereas shorter constructs were no longer affected. Down-regulation of HL-305 was only apparent at 72 h; at earlier time points, HL-305 activity was not significantly affected by overexpression of SREBP2 (83 ± 16% of control, n = 5). We conclude therefore, that the down-regulation of the HL promoter by SREBP2 is predominantly effected through the -325/-305 region.

**Figure 6:**
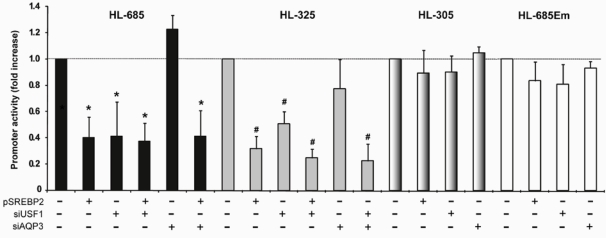
The effect of SREBP2 and USF1 is mediated by the -310 E-box of the HL gene. HepG2 cells were transfected with the indicated HL-promoter constructs without (-) or with (+) 6 ng pSREBP2 and/or 100 ng siUSF1 or siAQP3. Luciferase activity was determined after 48 h; data are expressed as fold increase relative to the transfection with the respective HL-promoter construct (n = 3–11; *: *P* < 0.05 vs. control of HL-685, ^#^: *P* < 0.05 vs. control of HL-325).

We have previously demonstrated the presence of a functional E-box at -307/-312 of the HL promoter region, which mediates transactivation by upstream stimulatory factors (USF) 1 and 2 [31]. To test the importance of this E-box and endogenous USF proteins in mediating the effect of SREBP2, we made an HL-685 construct in which the E-box was mutated (HL-685Em). In addition, we inhibited expression of endogenous USF1 by RNA interference (siUSF1). The activity of HL-685 and HL-325 was suppressed by approximately 60% by siUSF1 and SREBP2 alone (Figure 6). The effects of siUSF1 and SREBP2 were not additive. In contrast, an unrelated siRNA did not significantly affect HL promoter activity. Removal or mutation of the -307/-312 E-box in the HL-305 and HL-685m constructs, respectively, resulted in the almost complete loss of sensitivity to siUSF1 as well as SREBP2 (Figure 6). These findings strongly indicate that nSREBP2 exerts its effect through USFs and the E-box at -307/-312.

### 2.5. Nuclear USF1 Is Increased by Oleate and Reduced by nSREBP2 Overexpression

**Figure 7:**
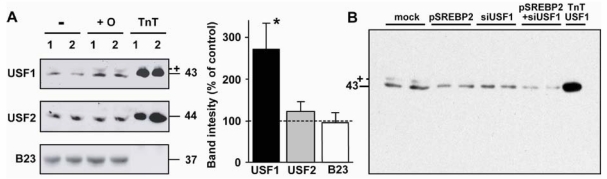
Effect of oleate and nSREBP2 on nuclear USF1 protein abundance. (A) HepG2 cells were incubated for 48 h in the absence (-) or presence (+O) of oleate. Thereafter, nuclear extracts were prepared and used for immunoblotting. B23 protein served as internal control. TnT 1 and 2 are 0.5 and 1.0 μL, respectively, of *in vitro* made USF protein (left panel). The data were quantified by densitometry and expressed as percentage of control (right panel; n = 3–4; *: *P* < 0.05 vs. control). (B) HepG2 cells were transfected without (mock) or with 100 ng pSREBP2, siUSF1, or both. Nuclear extracts were immunoblotted with anti-USF1. The molecular sizes are indicated in kDa. The bands marked with + may represent a modified form of USF1.

Nuclear extracts were prepared from HepG2 cells after incubation for 48 h with or without 1 mM of BSA-bound oleate. As shown in Figure 7A, the amount of USF1 was 2.7±0.6-fold higher in the oleate-treated cells (n = 3, *P* = 0.04), whereas USF2 expression was not significantly increased (1.2 ± 0.2-fold, n = 3). We have previously shown by chromatin immunoprecipitation assays that USF1 is bound to the proximal region of the human HL gene in HepG2 cells [31,32], that the degree of binding correlates with HL gene expression levels [31], and that co-transfection of HepG2 cells with USF1-expression plasmids results in strong upregulation of HL expression [23,31].

When cells were transfected with pSREBP2, the amount of USF1 protein was reduced to 45%–55% of control, similar to the effect seen with siUSF1 (Figure 7B). The amount of endogenous USF1 protein was further reduced to 15%–20% of untreated controls by the combined transfection with SREBP2 and siUSF1.

### 2.6. Discussion

Here we have demonstrated for the first time that the monounsaturated fatty acid oleate affects gene expression in liver cells by increasing the nuclear abundance of USF1. The USFs are ubiquitous transcription factors with a broad spectrum of target genes [29], in particular lipogenic and other insulin-responsive genes [30]. USF1 and USF2 are constitutively expressed, and their activity is modulated by reversible phosphorylation and acetylation [29,30,37,38]. In the regulation of the fatty acid synthase gene in liver cells, posttranslationally modified USF1 has recently been shown to be the sensor of nutritional status during fasting and refeeding [38]. Nevertheless, signalling through USF1 also occurs by regulating the nuclear abundance of USF1 [32,39,40]. Others have shown that upregulation of USF1 in HepG2 cells results in the elevated expression of several genes including apolipoproteins, angiotensinogen, and Cyp1A2. Upregulation of liver USF1 by oleate, and subsequent activation of lipogenic genes, would represent a feed-forward cycle leading to increased triglyceride and VLDL synthesis. Indeed, the USF1 gene has been implicated in the elevated plasma triglyceride trait of familial combined hyperlipidemia [30]. Here we showed that oleate increases HL expression in HepG2 cells. A high oleate supply to the liver results in increased production of VLDL. Increased HL activity has been proposed to facilitate VLDL production by supplying the liver with sufficient phospholipid precursors [13]. An increase in nSREBP2 activity reflects a low intracellular cholesterol status, and loss of cholesterol via VLDL may be reduced by the nSREBP2-mediated suppression of HL activity.

It is not clear how oleate increases nuclear USF1 protein. PUFAs have been shown to affect gene expression predominantly through PPARs and SREBPs. It is not likely that the USF1 gene is upregulated by PPARα, since USF1 target genes are predominantly lipogenic and PPARα targets are predominantly involved in lipid oxidation. Oleate reduces SREBP activity in HepG2 cells, and overexpression of nSREBP2 reduces expression of USF1 protein and its target gene, HL. Suppression of HL expression by SREBP2 is not only seen upon forced overexpression of nSREBP2, but also upon treatment with a statin [16,23,27]. We may argue therefore that the oleate-mediated lowering of SREBP2 increases USF1 protein and hence hepatic lipase expression, though this was not actually shown. Although oleate is a relatively poor suppressor of nSREBP1, the USF1-gene may also be a target of SREBP1. However, the HL gene is not suppressed by overexpression of nSREBP1 as would be predicted when SREBP1 would affect USF1. In addition, HL promoter activity is not significantly upregulated by treating HepG2 cells with PUFAs [41], which predominantly lower nSREBP1. On the other hand, when SREBP activity in the HepG2 cells was strongly suppressed by incubation with cholesterol+25-hydroxycholesterol, HL promoter activity was not significantly upregulated, suggesting that lowering of SREBP1 and SREBP2 is not sufficient to increase nuclear USF1. In addition, USF1 has not been identified as a direct SREBP target gene in the comparative mRNA screen performed by Horton *et al.* [34]. We propose therefore that treatment of HepG2 cells with oleate upregulates USF1 expression and hence HL expression independently of SREBPs. Further studies are required to elucidate how oleate affects USF1 expression.

In general, SREBP1 and SREBP2 activate transcription of their target genes, and the HL gene would have been one of a few genes that are suppressed by SREBP2. From their comparative mRNA screen, Horton *et al.* [34] dismissed the HL gene being a direct SREBP target. The delay between nSREBP2 expression and HL suppression is in line with this notion. Our data show that SREBP2 overexpression affects HL promoter activity mainly through the functional USF binding E-box at -307/-312. USF1 has been shown to bind to SREBP1 [38], but this only occurs when both transcription factors are simultaneously bound to DNA in close proximity. Therefore, the possibility that USF1 is prevented from binding to the proximal HL promoter by complex formation with SREBPs in solution appears unlikely. SREBPs bind to E-boxes as well as SREs, but in contrast to SREs, binding to E-boxes does not result in transactivation [42]. SREBP2 overexpression may also reduce HL expression by competing with USF1 for binding at the -310 E-box. Hence, other mechanisms may be involved in the inhibition of HL expression by nSREBP2, in addition to the lowering of USF1 expression.

## 3. Experimental

### 3.1. Cell Culture

HepG2 cells (ATCC, Rockville, MD, USA) were maintained in Dulbecco's Modified Eagle's Medium (DMEM; GIBCO-BRL, Breda, Netherlands), supplemented with 10% fetal calf serum (FCS), 50 IU/mL penicillin and 50 μg/mL streptomycin. The cells were kept at 37 °C in a humidified atmosphere of 5% CO_2_ in air. Twenty-four hours before the start of the experiment, the cells were plated at 30% confluence in 6- or 24-well culture plates (Nunc, Roskilde, Denmark).

Bovine serum albumin-bound oleate (molar ratio 1:6) was added to the medium to a final oleate concentration of 1 mM, which is the upper limit of free fatty acid concentrations found in human plasma [43]. The control medium contained less than 0.05 mM non-esterified fatty acids (NEFA C-kit, Wako Chemicals, Germany). In some experiments, the cells were incubated for 48h with 20 μg/mL cholesterol plus 2 μg/mL of 25-hydroxycholesterol (both from Sigma, St. Louis, MO, USA); additions were made from 1,000-fold stocks in ethanol. Unless indicated otherwise, the media including additions were refreshed after 24 h. For determination of hepatic lipase secretion, cells were grown in 6-wells plates and 2 mL medium/well. After 48 h incubation, the media were removed and the cells were incubated for an additional 12 h in 1 mL/well of fresh medium containing 25 IU heparin (Leo Pharmaceuticals, Breda, The Netherlands). Hepatic lipase activity was assayed in the cell-free media as described before [44]. Enzyme activity was expressed as mU (nmoles of free fatty acids released per min from triolein substrate).

Total cellular RNA was isolated using the Trizol reagent (Invitrogen, Breda, The Netherlands), and the amount of HL mRNA and HMG-CoA reductase (HMGR) mRNA was determined by reverse transcription followed by real-time PCR (RT-qPCR) using MyIQ from BioRad, as described previously [38]. Primers used for HMGR mRNA were 5’-GAA GCT GTC ATT CCA GCC A-3’ and 5’-GAA CTA CCA ACA TTC TGT GC-3’.

### 3.2. Plasmids

Recombinant DNA techniques were performed according to standard procedures [45]. Oligonucleotides were custom-made by Sigma (Cambridge, UK). Enzymes used were purchased from Roche (Basel, Switzerland). All inserts were verified by DNA sequencing (BaseClear, Leiden, The Netherlands).

A series of reporter plasmids was used containing different 5′-deletions of the parent human HL(−685/+13)-luc plasmid (further named HL-685), as described previously [23,31]. All inserts contained the same 3′-end (+13 relative to the transcription start site). In HL-685Em the E-box at -307/-312 (the -310 E-box) was mutated into a NheI site [31].

pSRE-luc containing the generic TATA-box and three SRE-elements of the hamster HMG-CoA synthase in pGL3-Basic was used as a SREBP-responsive reporter construct [23]. pSREBP1 and pSREBP2, containing the coding sequence of human nSREBP1 and nSREBP2 in pcDNA3.1, were kindly provided by B. Staels (Institut Pasteur, Lille, France). From pSREBP2, the ΔB-SREBP2 mutant was generated by deleting the 39bp fragment that codes for the basic DNA binding site (aa 331–343) [35,36]. The mutant was made by the PCR overlay technique [46] using SREBP2-specific forward and reverse primers of the sequence 5’-C CCC AAA GAA GGA GAA*TCC TCC ATC AAT GAC-3’ (* denotes the position of the 39bp to be removed) in combination with vector specific primers. After digestion with BamHI and XbaI, the PCR product was inserted into pcDNA3.1 (Invitrogen, Breda, The Netherlands).

RNA silencing of the USF1 gene was achieved with a shUSF1 plasmid as described previously [31,32]; shAQP3 plasmids against non-related AQP3 gene (a kind gift from B. Tilly, Biochemistry, Rotterdam) served as negative control. pRL-GAPDH, which contains the human glyceraldehyde 3‑phosphate dehydrogenase gene promoter in pRL-null, was kindly provided by A.A.F. de Vries (LUMC, Leiden, The Netherlands).

### 3.3. Promoter-Reporter Assays

Promoter reporter assays were performed in transiently transfected HepG2 cells, as described previously [23]. Cells were co-transfected with luciferase reporter constructs (0.4 μg/well), pRL‑GAPDH (20 ng/well) and the indicated amounts of pSREBP1, pSREBP2 or shUSF1, complemented with pcDNA3.1. After 3h, the media were refreshed and BSA-bound oleate or cholesterol+25-hydroxycholesterol was added when indicated. Media were refreshed again at 24 h. After 48 h, cell extracts were prepared with lysis buffer (Roche, Basel, Switzerland), and firefly and renilla luciferase activities were determined with the FireLight kit (Perkin-Elmer, Boston, MA, USA) and the Packard Top Count NXT luminometer. Luciferase activities were expressed as the ratio between firefly and renilla counts.

### 3.4. Protein Expression

Nuclear extracts were prepared at 48h post-transfection [32], and protein concentrations were determined by DC protein assay (Bio-Rad). Alternatively, USF and SREBP2 proteins were expressed in vitro using the TnT T7 Quick Coupled Transcription/Translation system (Promega) with the appropriate expression vectors as DNA template. Nuclear extracts (20 μg) and TnT expression mixtures (0.5–1 μL) were separated by SDS-PAGE on 10% gels, and transferred to nitrocellulose membrane (Hybond ECL; Amersham Biosciences, Amersham, UK). After blocking overnight with 5% milk powder in TBS (20 mM Tris pH 7.6 in 150 mM NaCl), the membranes were incubated with either rabbit polyclonal anti-human SREBP2 (RS004, kindly provided by R. Sato, University of Tokyo, Japan [47]; 1:500 dilution in 5% milk powder/0.05% Tween-20 in TBS), mouse monoclonal anti-SREBP2 (IgG-1D2 hybridoma supernatant (ATCC, Rockville, MD, USA [48]; 1:50 dilution in 0.5% milk powder/0.05% Tween-20 in TBS), or a 1:4,000 dilution of rabbit polyclonal anti-human USF1 or USF2 (SC-229X and SC-862X, respectively; Santa Cruz Biotechnology, Santa Cruz, CA, USA; 1:4,000 dilution in 0.5% milk powder/0.05% Tween-20 in TBS). Subsequently, the blots were incubated for 1h with horseradish peroxidase-conjugated donkey anti-rabbit IgG (Amerham Biosciences, UK) or goat anti-mouse IgG (Promega), diluted 1:2,500 in 0.5% milk powder/0.05% Tween-20 in TBS. The secondary antibody was visualized by enhanced chemiluminescence (Super Signal West Pico Chemiluminescent Substrate, Pierce, Rockford, IL, USA) and exposure to Hyper ECL film (Amersham Biosciences, UK). The images were quantified by densitometry using the GS-800 Calibrated Densitometer from BioRad.

### 3.5. Statistics

Data are expressed as means ± sd. Statistical significances were determined by Student’s t-test.

## 4. Conclusions

We have shown here that oleate increases the nuclear abundance of USF1 in human hepatoma cells. Together with previous studies, this suggests that oleate affects expression of the HL gene through USF1. USF1 may be elevated by oleate secondary to suppression of nuclear SREBP2 activity, but our data suggests that oleate affects USF1 independently of SREBP2. Hence, USFs may represent an additional class of transcription factors, besides SREBPs and PPARS, through which fatty acids affect mammalian gene expression. USF1 has been shown to be a sensor of nutritional status during fasting and refeeding in the liver [38]. Our findings suggest that USF1 may function as a sensor of fatty acid supply to the liver. 
